# Ambroxol Upregulates Glucocerebrosidase Expression to Promote Neural Stem Cells Differentiation Into Neurons Through Wnt/β-Catenin Pathway After Ischemic Stroke

**DOI:** 10.3389/fnmol.2020.596039

**Published:** 2021-01-20

**Authors:** Hongfei Ge, Chao Zhang, Yang Yang, Weixiang Chen, Jun Zhong, Xuanyu Fang, Xuheng Jiang, Liang Tan, Yongjie Zou, Rong Hu, Yujie Chen, Hua Feng

**Affiliations:** ^1^Department of Neurosurgery and Key Laboratory of Neurotrauma, Southwest Hospital, Third Military Medical University (Army Medical University), Chongqing, China; ^2^Department of Emergency, Affiliated Hospital of Zunyi Medical University, Zunyi, China; ^3^Department of Neurosurgery, Hospital of People's Liberation Army, Nanchang, China

**Keywords:** ambroxol, neural stem cells, differentiation, Wnt/β-catenin pathway, ischemic stroke

## Abstract

Ischemic stroke has been becoming one of the leading causes resulting in mortality and adult long-term disability worldwide. Post-stroke pneumonia is a common complication in patients with ischemic stroke and always associated with 1-year mortality. Though ambroxol therapy often serves as a supplementary treatment for post-stroke pneumonia in ischemic stroke patients, its effect on functional recovery and potential mechanism after ischemic stroke remain elusive. In the present study, the results indicated that administration of 70 mg/kg and 100 mg/kg enhanced functional recovery by virtue of decreasing infarct volume. The potential mechanism, to some extent, was due to promoting NSCs differentiation into neurons and interfering NSCs differentiation into astrocytes through increasing GCase expression to activate Wnt/β-catenin signaling pathway in penumbra after ischemic stroke, which advanced basic knowledge of ambroxol in regulating NSCs differentiation and provided a feasible therapy for ischemic stroke treatment, even in other brain disorders in clinic.

## Introduction

Ischemic stroke has been becoming one of the leading causes resulting in mortality and adult long-term disability worldwide, and imposed heavy socioeconomic burden (George and Steinberg, 2015; Prabhakaran et al., 2015; Petro et al., 2016; Koh and Park, 2017; Yang et al., 2019). Once ischemia ictus, it always creates an irreversible infarct core resulting from rapid neural cell death of parenchyma in the ischemic epicenter (Petro et al., 2016). While, surrounding the infarct core, also known as the penumbral area, could be rescued with timely medical intervention, including intravenous thrombolysis and mechanical thrombectomy, during the first few hour post-ischemia (Prabhakaran et al., 2015; Xiong et al., 2019). Unfortunately, only a small portion (~5%) of patients are received timely therapies. Herein, exploring the feasible therapeutic strategies to diminish penumbra deterioration or to restore the penumbra has aroused great attention for researchers after ischemic stroke. Cumulative evidence has indicated that activation of endogenous neural stem cells (eNSCs), which are specifically resided in the dentate gyrus (DG) and subventricular zone (SVZ), could proliferate *in situ*, migrate toward penumbra, and differentiated into three main neural subtype cells, benefiting penumbra rehabilitation after ischemic stroke (Hao et al., 2014; Huang and Zhang, 2019; Yang et al., 2019; Dillen et al., 2020). However, previous studies also reveal that the majority of eNSCs migrated from SVZ differentiate into astrocytes forming glial scar in penumbra after brain injury (Hermann et al., 2014; Grégoire et al., 2015). Hence, developing methods to promote eNSCs differentiation into neurons, instead of astrocytes, in peri-infarct regions might be a feasible therapeutic strategy for the treatment of ischemic stroke.

Ambroxol might serve as a neuroprotective target, except for enhancing pulmonary ventilation dysfunction to fight against post-stroke pneumonia. Post-stroke pneumonia, which is associated with a 49% increase in 1-year mortality, is also the most frequent complication in patients with ischemic stroke (de Montmollin et al., 2019). Except for administration of antibiotics for pulmonary infection, ambroxol, a small molecule chaperone, usually serves as an effective airway humidification liquid, which could improve lung function not only *via* facilitating the synthesis and secretion of pulmonary surfactant, but also through diluting sputum, thereafter, to promote pulmonary ventilation dysfunction and to inhibit further pulmonary damage, finally to enhance functional recovery after severe brain injury (Su et al., 2016). Thereafter, ambroxol is often applied to treat post-stroke pneumonia after ischemic stroke. Subsequently, previous studies have demonstrated that ambroxol could easily and effectively cross the blood brain barrier (BBB) and has no adverse effect even at high dose (Migdalska-Richards et al., 2016, 2017; Mullin et al., 2020). Furthermore, previous researches have revealed that ambroxol therapy is safe and well-tolerated, and might provide a potential novel target for the treatment of Parkinson disease from bench to bedside (Migdalska-Richards et al., 2016; Silveira et al., 2019; Mullin et al., 2020). Most recently, our previous study represents that 35 mg/kg and 70 mg/kg ambroxol promotes neuronal survival and decreases white matter fiber bundle damage *via* mitigating microglial activation and reducing proinflammatory cytokine accumulation in mice with intracerebral hemorrhage (ICH) (Jiang et al., 2020). Above evidence suggests that ambroxol hold neuroprotective ability, except for fighting against post-stroke pneumonia. Hence, the effect of ambroxol on penumbra and potential mechanism need to be further investigated.

Glucocerebrosidase (GCase) might activate Wnt/β-Catenin signaling pathway to regulate NSCs differentiation induced by ambroxol. Previous studies have certified that administration of ambroxol could increase the brain GCase activity not only in healthy non-human primates (Migdalska-Richards et al., 2017), but also in Parkinson's disease to improve pathological symptoms (Silveira et al., 2019; Mullin et al., 2020). Recently, research has indicated that GCase deficiency impedes neuronal progenitor cells (NPCs) differentiation into dopaminergic (DA) neurons through downregulation of canonical Wnt/β-catenin signaling in Gaucher's disease (GD) (Awad et al., 2017), implying that GCase upregulation could direct NSCs differentiation into neurons via activating canonical Wnt/β-catenin signaling pathway.

The canonical Wnt/β-catenin signaling pathway is a pivotal pathway that plays an evident role in promoting tissue repair and regulating stem cell activity, including NSCs proliferation and differentiation, when activated after brain injury (Ding et al., 2011; Arredondo et al., 2020; Marchetti et al., 2020). Meanwhile, Studies also represent that elevated β-catenin expression via activating Wnt/β-catenin signaling pathway not only regulates NSCs proliferation and differentiation, but also promotes neuron survival in cerebral diseases, including Parkinson's disease, Alzheimer's disease, and ischemic stroke (Marchetti, 2018; Qiu et al., 2019; Zhang et al., 2019; Arredondo et al., 2020). Furthermore, previous reports have indicated that Wnt/β-catenin signaling pathway and its downstream effectors are associated with neuroinflammation to regulate demyelination and remyelination in developing brain and Multiple Sclerosis (MS) (Vallée et al., 2018; Van Steenwinckel et al., 2019). In addition, Bianca Marchetti and Stefano Pluchino has summarized the role of the intrinsic Wnt/neuroinflammatory response in balancing the homeostasis of NSCs and neural progenitor cells (NPCs) in neurodegeneration and neurogenesis, as well as the crosstalk among mature neuron, astrocyte and microglia to modulate inflammatory response after centra nervous system (CNS) injury, such as traumatic injuries, PD and stroke (Marchetti and Pluchino, 2013). Hence, the role of Wnt/β-catenin signaling pathway in ambroxol serving as a potential neuroprotective agent is worthy of investigating.

In the present study, we hypothesized that ambroxol might facilitate NSCs differentiation into neurons via increasing GCase expression to activate Wnt/β-catenin signaling pathway, thereby benefited functional recovery in mice post-ischemia. The aim of the present study is to open a window for the use of ambroxol in treatment of ischemic stroke, and to provide basic knowledge and feasible target for cell replacement therapy in regulating NSCs differentiation into neurons, instead of astrocytes.

## Materials and Methods

### Mouse Distal Middle Cerebral Artery Occlusion (dMCAO) Model and Treatment

This study was approved by The Third Military Medical University Ethics Committee (approval no. SYXK 2012-0002) and all procedures were performed in accordance with the Chinese Animal Welfare Legislation for protection of animals used for scientific purposes. The model was established using distal middle cerebral artery occlusion (dMCAO) as previously described (Wang et al., 2016). A total of 68 male mice (4-week-old, 22–25 g, 60 mice used for research and 8 mice died during experiment) were provided by the Animal Experimental Center of Third Military Medical University and used in the present study. All mice were anesthetized with isoflurane/air mixture (2 l/min for induction and 1 l/min for maintenance). All surgical procedures were carried out under sterile condition and body temperature was maintained at 37 ± 0.3°C using a feedback-controlled heating system (Zhongshi, Inc., Beijing, China) during surgery. Briefly, a 4 mm horizontal incision in the skin between the left orbit and the auditory canal was performed under surgical microscope after depilation and disinfection with ethanol. Then, a 2 mm diameter hole using a micro drill was directly created over the middle cerebral artery (MCA). Afterward, the MCA was permanently cauterized at a point downstream of the lenticulostriate branches with a small vessel cauterizer after meninges were removed using forceps. For mice in sham group, the MCA were just exposed after meninges were removed using forceps. Subsequently, the muscles, subcutaneous tissue and skin were separately sutured. All mice were housed on a constant photoperiod (12-h light/dark cycle), temperature (22–25°C) and moisture (55–60%), and provided food and water *ad libitum* after surgery. Various concentration of ambroxol (Sigma-Aldrich, St. Louis, MO) was dissolved in normal saline and was intraperitoneally injected for 12 days (once a day, from day 1 to day 12) for behavioral tests, and 7 days (once a day, from day 1 to day 7) for immunostaining and immunoblot after dMCAO. For mice in group sham and dMCAO + Vehicle, they only received an equal volume of normal saline as ambroxol groups at the same time point.

### Behavioral Tests

For behavioral tests, Corner test, Beam walking test and Rotarod test were pre-operatively performed and then on days 1, 3, 7, 14, 21, and 28 post-dMCAO, respectively. All experiments and analyses were carried out by individual investigator blinded to treatment groups, and detailed procedures were described as follows.

Corner test was performed to assess the neurological impairment as previously described (Yang et al., 2019). Briefly, mice were placed half way between two angled boards with a 30-degree corner, and the head was oriented toward the corner. The turns in one vs. the other direction were recorded from ten trials for each mouse. Turning movements that were not part of a rearing movement are not scored. The percentage of turning right was calculated when they exited the corner.

Beam walking test was carried out to analyze the ability of maintaining balance, as previously described (Yang et al., 2018). Briefly, mice were trained 1 day before dMCAO, and only mice whose paws slipped down the horizontal surface of the beam (foot faults) fewer than 10 times per 50 steps were used for experiments. Then, mice were placed in one corner of the narrow beam and allowed to walk across the narrow beam from one end to the other for at least three times. The number of contralateral forelimb and hindlimb foot faults within 50 steps were recorded and analyzed, and mice that walked fewer than 50 steps after dMCAO were excluded.

Rotarod test was used to evaluate fore- and hind limb motor coordination and balance as described by our previous work (Yang et al., 2018; Jiang et al., 2020). The speed was initially set at 5 rpm and gradually increase to 35 rpm, and the latency to fall (or cling to and spin with the rod for 3 full rotations) within 3 min was recorded for further analysis. Three independent experiments for each mouse were separately conducted for at least 10 min interval. Every mouse was tested 1 day before establishing dMCAO model, and mice were ruled out if the latency was <60 s.

### 2, 3, 5-Triphenyltetrazolium Hydrochloride (TTC) Staining

TTC staining was applied for visualizing hypoxic brain tissue and for defining the size of cerebral infarction on day 7 following dMCAO, as previously described (Xiong et al., 2004; Yang et al., 2019). In short, brain samples were rapidly dissected after anesthetization with 2% isoflurane/air mixture (1–2 l/min), then coronally sectioned at 1 mm intervals. Afterward, the brain slices were incubated in 2% (w/v) 2,3,5-triphenyl tetrazolium hydrochloride (TTC, Sigma-Aldrich, St. Louis, MO) solution for 10 min at 37°C. Thereafter, the slices were fixed in 4% paraformaldehyde dissolved in phosphate buffered solution (PBS, 0.1 M, pH ~7.4) for 24 h at 4°C and images were captured using digital camera. Infarction area was determined by subtracting the normal area in the ischemic hemisphere from the area of the non-ischemic hemisphere based on TTC staining. Infarct volume was calculated by summing infarction areas of all sections and multiplying by slice thickness. All experiments and analyses were conducted by individual investigator blinded to treatment groups.

### Primary Mouse Neural Stem Cells (NSCs) Culture

A total of 11 E14.5 C57BL/6 mice were obtained from Animal Experimental Center of Third Military Medical University and applied for cultivating primary NSCs in the present study, as previously described (Ge et al., 2015, 2016; Yang et al., 2019). Briefly, the neocortices around lateral ventricle (LV) were dissected from pups for primary NSCs isolation after the embryonic mice were euthanized with rapid decapitation. Then, samples were washed twice in Dulbecco's modified Eagle's medium (DMEM; Hyclone, Logan, Utah) supplemented with 10% fetal bovine serum (FBS, vol/vol, Hyclone, Logan, Utah) after immersion in 0.25% trypsin-EDTA (Hyclone, Logan, Utah) for 30 min at 37°C. Then, the samples were triturated using a fire-polished Pasteur pipette and passed through a 100-μm Nylon cell strainer (BD Falcon, San Jose, CA) after they were rinsed twice with DMEM. Subsequently, cell suspensions were incubated in DMEM/F12 medium (Hyclone, Logan, Utah) supplemented with B27 (Gibco, Grand Island, NY), 20 ng/ml epidermal growth factor (EGF, Peprotech, Rocky Hill, NJ) and 20 ng/ml fibroblast growth factor-basic (bFGF, Peprotech, Rocky Hill, NJ) under humidified 5% CO_2_ condition at 37°C as recommended. For passaging cells, neurospheres were collected by centrifugation (300 rpm), then dissociated in StemPro Accutase Cell Dissociation Reagent (Gibco, Grand Island, NY) and incubated in the aforementioned medium. NSPCs, used for all experiments in the present study, were from passage 3 to 5.

### Oxygen Glucose Deprivation (OGD) Model

Oxygen glucose deprivation (OGD) model is widely used as a feasible *in vitro* model for ischemic stroke (Yang et al., 2019; Poupon-Bejuit et al., 2020). Hence, the OGD model was applied to examine the underlying potential cellular mechanisms in the present study. For OGD, the DMEM/F12 medium was replaced with glucose-free DMEM medium. Then, NSCs were incubated in a well-characterized and finely controlled ProOx-C-chamber system (Biospherix, Redfield, NY) with decreased O_2_ and increased N_2_ levels under humidified 5% CO_2_ condition at 37°C for 8 h. The O_2_ concentration in the chamber was controlled by the ProOx model 110 and maintained at 1%. For control group, NSCs were subjected to the same procedures as the OGD groups except that they were exposed to 21% O_2_ and normal glucose condition during the entire duration.

### NSC Differentiation

For differentiation, NSCs (1 × 10^5^/ml) were seeded in 10 μg/ml poly-L-ornithine (PLO)-pre-coated confocal culture dishes and incubated in DMEM/F12 medium supplemented with B27 and 1% GlutaMAX™ with or without Ambroxol for 10 days under humidified 5% CO_2_ condition at 37°C. Cardamonin (cat. no. 19309-14-9, Sigma-Aldrich, Munich, Germany) and XAV939 (10 nM, cat. no. 284028-89-3, Sigma-Aldrich, Munich, Germany) were dissolved in DMSO in the stock solution (10 mM), and diluted in culture medium at the final concentration. Recombinant mouse β-glucocerebrosidase (GBA, 10 ng/ml, cat. no. ab235724, Abcam, Cambridge, UK) and various concentration of ambroxol were dissolved in PBS in the stock solution. All chemicals were added to the culture medium when NSCs exposed to OGD for 10 days during differentiation. For NSCs in group OGD + Vehicle, they received an equal volume of PBS and DMSO (0.1%) at the same time point as OGD groups. The culture medium was exchanged every 2–3 days.

### Bromodeoxyuridine (BrdU) Injection

To tell the difference of DCX^+^ and GFAP^+^ cells between differentiation from NSCs, which included proliferated *in situ* or migrated from subventricular zone (SVZ), and local epibiotic neural lineage cells, mice were received two intraperitoneal BrdU injections (50 mg/kg, dissolved in normal saline) per day for 3 consecutive days, then mice were killed 4 days after the last injection.

### Immunostaining

For immunostaining, NSCs (1 × 10^5^/ml) cultured in poly-L-ornithine (PLO)-pre-coated confocal culture dishes or frozen brain sections (−20°C) from each group were post-fixed using 4% paraformaldehyde (PFA) in 0.01 M phosphate buffer saline (PBS) for 1 h at room temperature after rinsed twice with PBS. Then, the samples were blocked with 10% normal goat serum supplemented with 0.5% Triton X-100 (Sigma-Aldrich, Munich, Germany) in PBS for 30 min at room temperature. Afterward, samples were immersed in rabbit polyclonal anti-GBA (1:100, cat. no. ab175869, Abcam, Cambridge, UK), mouse monoclonal anti-MAP2 (1:100, cat. no. ab11268, Abcam, Cambridge, UK), rat monoclonal anti-GFAP (1:300, cat. no. 13-0300, Thermo Fisher Scientific, Waltham, MA, USA), mouse monoclonal anti-β-catenin (1:100, cat. no. ab19381, Abcam, Cambridge, UK), mouse monoclonal anti-BrdU (1:100, cat. no. MAB4072, Millipore, Darmstadt, Germany), rabbit polyclonal anti-DCX (1:100, cat. no. ab18723, Abcam, Cambridge, UK), rabbit polyclonal anti-GFAP (1:100, cat. no. ab53554, Abcam, Cambridge, UK), mouse monoclonal anti-DCX (1:100, cat. no. ab135349, Abcam, Cambridge, UK), mouse monoclonal anti-Nestin (1:100, cat. no. ab6142, Abcam, Cambridge, UK) or Alexa Fluor® 647-conjugated mouse monoclonal anti-Nestin (1:100, cat. no. 655107, Biolegend, San Diego, USA) overnight at 4°C. Then, samples were incubated with Alexa Fluor® 555 or 488-conjugated secondary antibody (1:100; cat. nos. A0453 and A0423; Beyotime Institute of Biotechnology, Beijing, China) at room temperature for 1 h. Cell nuclei were counterstained with 4′,6-diamidino-2-phenylindole (DAPI, Sigma-Aldrich, Munich, Germany) for 10 min at room temperature. Subsequently, samples were mounted onto glass slides and images were captured by a confocal microscope (LSM780; Carl Zeiss, Weimar, Germany) and evaluated using Zen 2011 software (Carl Zeiss, Weimar, Germany).

For BrdU immunostaining, frozen brain sections were firstly incubated in 2 N HCl at for 30 min 37°C, then rinsed with 0.1 M borate solution (pH 8.5) twice for 10 min. Afterward, they were incubated in 3% H_2_O_2_ for 30 min, and blocked with 5% normal goat serum for 1 h at room temperature. Then, the immunostaining was performed according to the above procedures.

For immunostaining analyzing, the samples were obtained from penumbra (~1–2 mm among infarct core as shown in Figure 3A). For each sample, six sections were immunostained, analyzed, and the cross-sectional areas were calculated and reported as the average of four independent measurements. All measurements were performed by an individual investigator who was blinded to the experiment groups.

### Immunoblotting

Brain tissues from penumbra or cell samples were homogenized in 200 μl ice-cold RIPA (Beyotime Institute of Biotechnology, Beijing, China) supplemented with protease inhibitor cocktail (Roche, Indianapolia, IN, USA). Then, lysate was collected after centrifugation at 10,000 g at 4°C for 20 min. Afterward, the protein concentration was determined by an enhanced BCA Protein Assay Kit (Beyotime, Beijing, China). Proteins (50 μg for brain tissue and 20 μg for cell sample) were separated by 10% SDS-PAGE under reducing conditions and electro-blotted to polyvinylidene difluoride (PVDF, Roche, Indianapolia, IN, USA) membranes. Then, the membranes were immersed in 5% (w/v) non-fat dry milk (Beyotime Institute of Biotechnology) in TBS with Tween-20 (TBST) at room temperature for 2 h. Afterward, the membranes were cut out at different parts according to a pre-stained protein molecular ladder (cat. no. 26616; Thermo Fisher Scientific, Inc.) to allow separate detection of proteins migrating at the same distance, and were incubated in primary antibodies, rabbit polyclonal anti-GBA (1:1,000, cat. no. ab175869, Abcam, Cambridge, UK), mouse monoclonal anti-β-catenin (1:1,000, cat. no. ab19381, Abcam, Cambridge, UK), rabbit polyclonal anti-β-catenin (1:1,000, cat. no. ab16051, Abcam, Cambridge, UK), rabbit polyclonal anti-phospho-β-catenin (1:1,000, cat. no. 4,176, Cell Signaling Technology, Danvers, MA, USA), rabbit polyclonal anti-non-phospho (active)-β-catenin (1:1,000, cat. no. 19,807, Cell Signaling Technology, Danvers, MA, USA), rabbit monoclonal anti-Axin2 (1:1,000, cat. no. BM5071, Boster, Wuhan, China), rabbit polyclonal anti-DCX (1:100, cat. no. ab18723, Abcam, Cambridge, UK), rabbit polyclonal anti-GFAP (1:100, cat. no. ab53554, Abcam, Cambridge, UK) or mouse monoclonal anti-GAPDH (1:1,000; cat. no. AF0006; Beyotime Institute of Biotechnology) overnight at 4°C. Subsequently, the membrane was incubated in corresponding horseradish peroxidase (HRP)-conjugated secondary antibody after rinsed twice with TBST. All membranes were visualized by a ChemiDoc™ XRS^+^ imaging system (Bio-Rad, California, USA) using the WesternBright ECL Kits (Advansta, Menlo Park, CA, USA). Densitometric measurement of each membrane was performed using Image Lab™ software (Bio-Rad, California, USA).

### Reverse Transcription-Quantitative Polymerase Chain Reaction (RT-qPCR)

After the NSCs were exposed to different concentration of ambroxol under OGD for 8 h, the total RNA was extracted using TaKaRa MiniBEST Universal RNA Extraction Kit (TaKaRa, Tokyo, Japan) according to the manufacturer's instructions. Then, 1 μg RNA was reverse transcribed into cDNA using PrimeScript RT reagent Kit with gDNA Eraser (cat. no. RR0047A, TaKaRa, Tokyo, Japan) according to the manufacturer's instructions. Subsequently, qPCR was performed using CFX96 System (Bio-Rad, CA, USA) with SYBR Premix Ex TaqII (Tli RNaseH Plus) (cat. no. RR820A, TaKaRa, Tokyo, Japan) under the following condition: 95°C for 30 s, 40 cycles at 95°C for 5 sec and 60°C for 30 s. Relative mRNA levels were normalized to GAPDH and analyzed using the 2^−ΔΔCq^ method. Primer sequences used in the present study were as follows:

**Table:** 

	**Forward**	**Reverse**
Axin2	5′-TGACTCTCCTTCCAGATCCCA-3′	5′-TGCCCACACTAGGCTGACA-3′
GBA	5′- GCCAGGCTCATCGGATTCTTC-3′	5′-CACGGGGTCAAGAGAGTCAC-3′
β-catenin	5′-ATGGAGCCGGACAGAAAAGC-3′	5′-CTTGCCACTCAGGGAAGGA-3′
GAPDH	5′-AACGGGAAGCCCATACC-3′	5′-CATACTCAGCACCGGCCTCA-3′

### Statistical Analysis

The statistical analysis was carried out using SPSS v19.0 (SPSS Inc., Chicago, IL). Comparisons between two groups were analyzed using Student's t tests or analysis of variance (ANOVA), followed by Tukey's *post hoc* test in case of the data with a normal distribution using a Shapiro–Wilk normality test. Moreover, data failing the normality test were represented as median and interquartile range (IQR) analyzed using the Mann–Whitney U test. A *p* < 0.05 was considered as significant difference.

## Results

### Ambroxol Facilitates Functional Recovery and Reduces Infarct Volume in Mice After dMCAO

**Figure 1:**
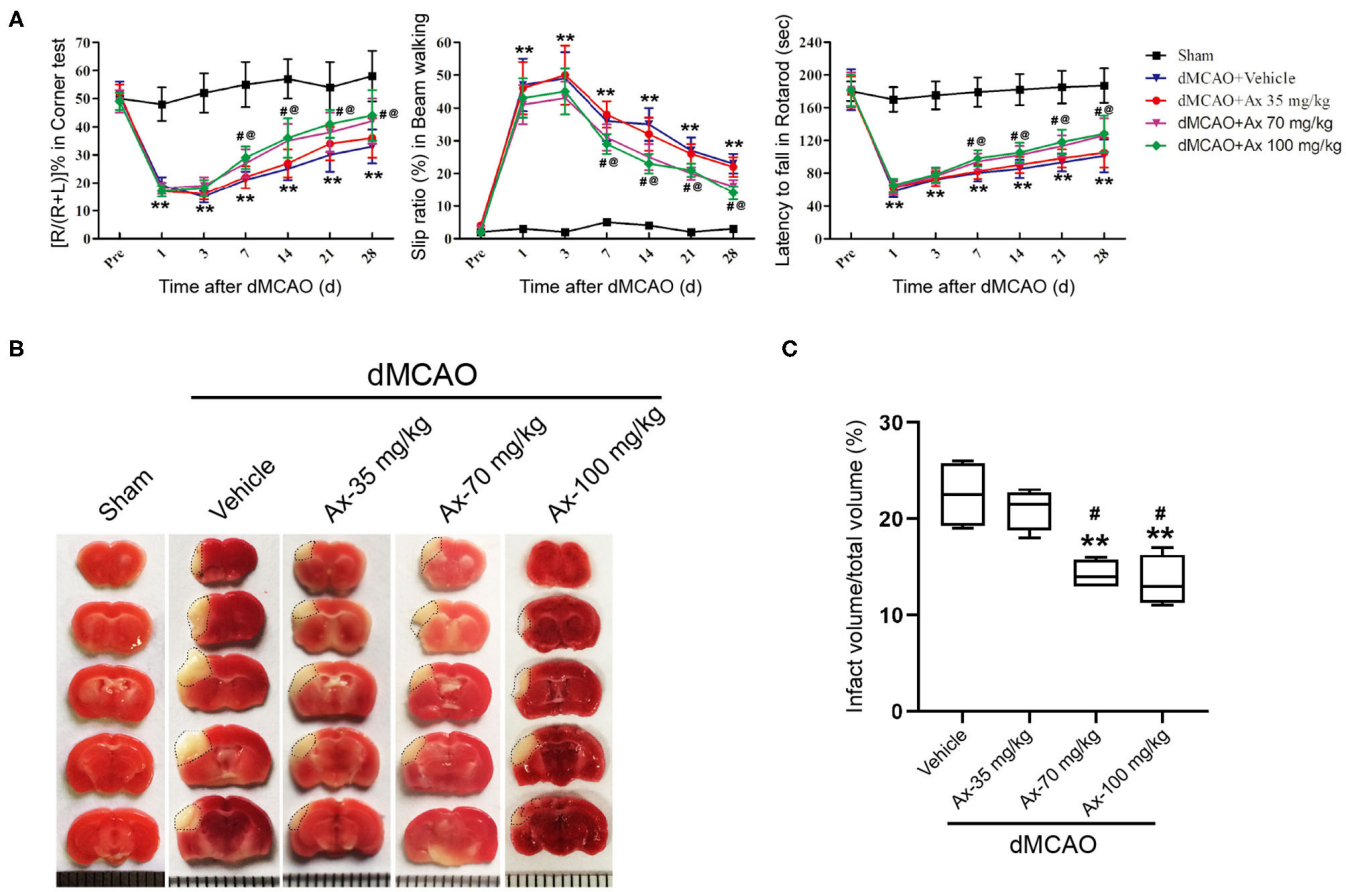
Ambroxol (Ax) facilitates functional recovery and reduces infarct volume in mice after dMCAO. **(A)** Summarized data showing behavioral tests in different groups on day 1, 3, 7, 14, 21, and 28 after dMCAO. **P < 0.01 vs. control group; ^#^*P* < 0.05 vs. dMCAO + Vehicle; ^@^*P* < 0.05 vs. dMCAO + Ax-35 mg/kg. (**B)** Representative TTC staining images in various groups. White area with black dotted showed infarct area. (**C)** Quantitative data of infract volume in various groups on day 7 following dMCAO. **P < 0.01 vs. dMCAO + Vehicle group; ^#^*P* < 0.05 vs. dMCAO + Ax-35 mg/kg. Ax, ambroxol; dMCAO, distal middle cerebral artery occlusion.

Based on our previous investigation that 35 mg/kg and 70 mg/kg ambroxol could facilitate neuronal survival and decrease white matter fiber bundle injury by suppressing microglial activation and decreasing proinflammatory cytokine accumulation in mice with intracerebral hemorrhage (ICH), finally enhance functional recovery (Jiang et al., 2020). Here, to explore the effect of administration of ambroxol on functional recovery after ischemic stroke, three concentrations of ambrxol (35, 70, 100 mg/kg) were applied to assess the feasible therapeutic concentration. The results indicated that mice in groups 70 and 100 mg/kg ambroxol obtained better functional recovery, including Corner, Beam walking and Rotarod tests, compared with that in groups Vehicle and 35 mg/kg ambroxol (Figure 1A). To further uncover the reason why administration of 70 and 100 mg/kg ambroxol potentiates functional recovery, the infarct volume on day 7 was calculated using TTC staining. Our results revealed that administration of 70 and 100 mg/kg ambroxol markedly reduced the infarct volume, in comparison with that in Vehicle and 35 mg/kg ambroxol groups (Figures 1B,C). Mechanistically, these results delineated that administration of 70 mg/kg and 100 mg/kg enhanced functional recovery by virtue of decreasing infarct volume. Herein, the proper therapeutic dosage of ambroxol used in the present study was 70 mg/kg in *in vivo* experiment.

### Administration of 70 mg/kg Ambroxol Promotes NSCs Differentiation Into Neurons and Inhibits NSCs Differentiation Into Astrocytes in Penumbra After dMCAO

**Figure 2:**
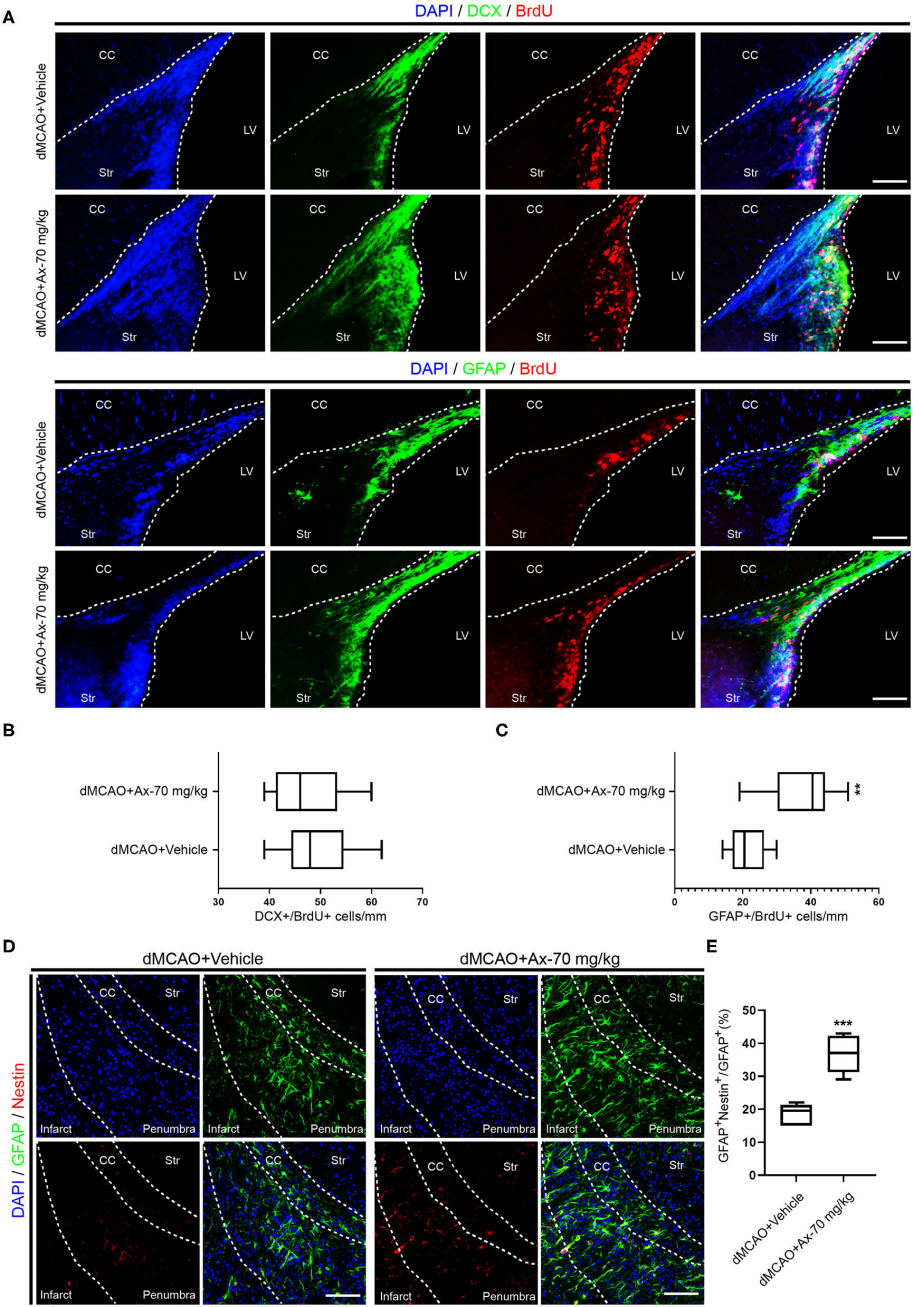
Administration of 70 mg/kg Ax enhances GFAP^+^ cells proliferation in SVZ and increases the portion of NSCs in penumbra after dMCAO. **(A)** Representative immunostaining images showing colocalization of BrdU (red) with DCX (green) or GFAP (green) in SVZ. Scale bar: 20 μm. **(B)** Quantitative analysis of the average number of BrdU^+^ and DCX^+^ cells in SVZ in group dMCAO + Vehicle and dMCAO + Ax-70 mg/kg. **(C)** Quantification of the average number of colocalization of BrdU (red) and GFAP (green) in SVZ. **P < 0.01 vs. dMCAO + Vehicle group. **(D)** Typical immunostaining images showing colocalization of Nestin (red) with GFAP (green) in penumbra. Scale bar: 20 μm. **(E)** Quantitative analysis of the percentage of Nestin^+^ and GFAP^+^/GFAP^+^ in penumbra in group dMCAO + Vehicle and dMCAO + Ax-70 mg/kg. ***P < 0.001 vs. dMCAO + Vehicle group. Ax, ambroxol; dMCAO, distal middle cerebral artery occlusion; GFAP, glial fibrillary acidic protein; CC, corpus callosum; Str, striatum; LV, lateral ventricle.

**Figure 3:**
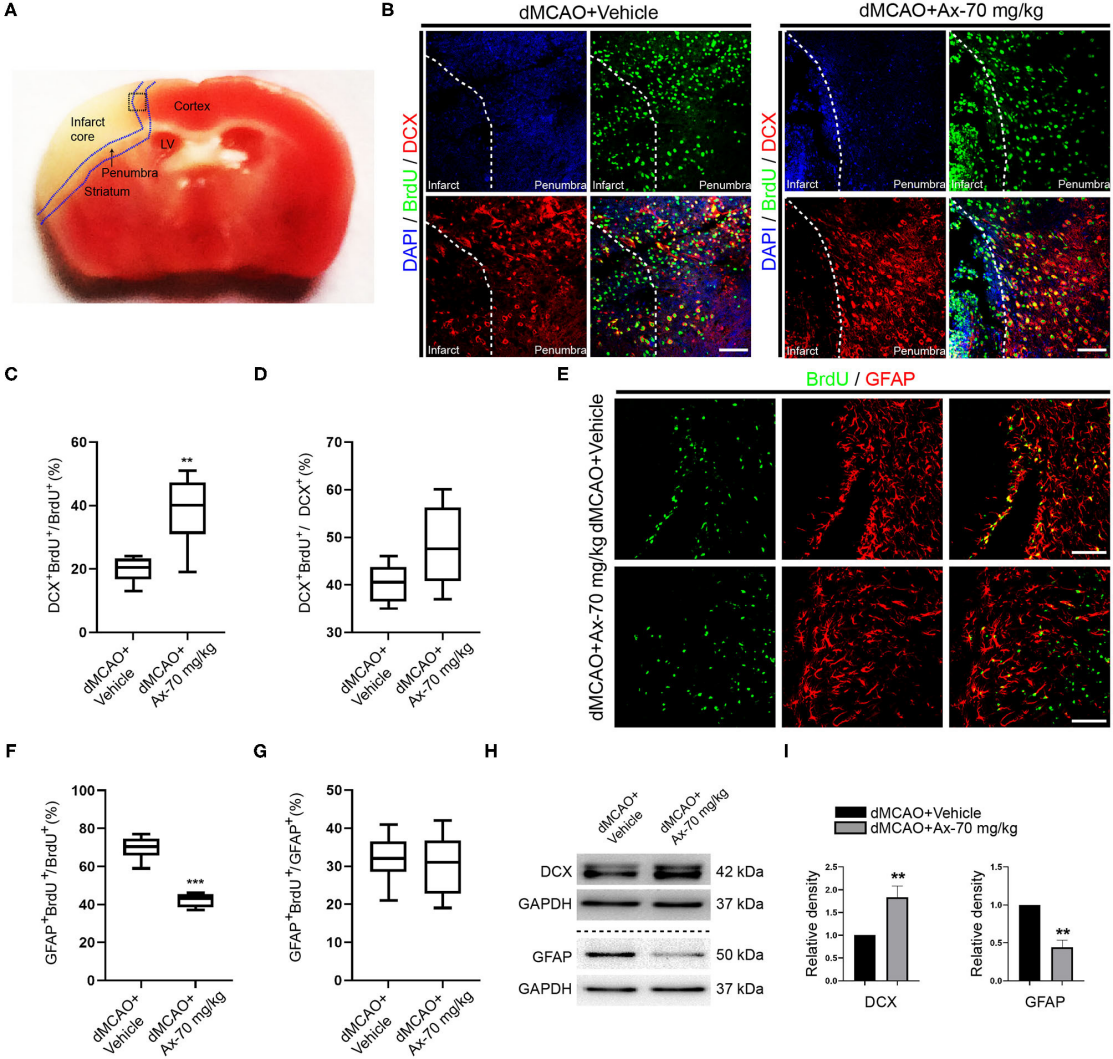
Administration of 70 mg/kg Ax promotes NSCs differentiation into neurons and inhibits NSCs differentiation into astrocytes in penumbra after dMCAO. **(A)** Schematic illustration showing the regions after dMCAO: penumbra in blue dotted line; the black dotted square indicating region of interest (ROI) for immunostaining. **(B)** Representative immunostaining images showing colocalization of BrdU (green) and DCX (red) in ROI. DAPI was applied to counterstain the nuclei. Scale bar: 20 μm. **(C)** Quantitative analysis of the percentage of DCX^+^ and BrdU^+^/BrdU^+^ in group dMCAO + Vehicle and dMCAO + Ax-70 mg/kg. **P < 0.01 vs. dMCAO + Vehicle group. **(D)** Quantitative analysis of the percentage of DCX^+^ and BrdU^+^/DCX^+^ in group dMCAO + Vehicle and dMCAO + Ax-70 mg/kg. **(E)** Typical immunostaining images showing colocalization of BrdU (green) and GFAP (red) in ROI. DAPI was applied to counterstain the nuclei. Scale bar: 20 μm. **(F)** Quantitation of the portion of GFAP^+^ and BrdU^+^/BrdU^+^ in group dMCAO + Vehicle and dMCAO + Ax-70 mg/kg. ***P < 0.001 vs. dMCAO + Vehicle group. **(G)** Quantitative analysis of the percentage of GFAP^+^ and BrdU^+^/GFAP^+^ in group dMCAO + Vehicle and dMCAO + Ax-70 mg/kg. **(H)** Immunoblot bands illustrating the expression of DCX and GFAP in penumbra in group dMCAO + Vehicle and dMCAO + Ax-70 mg/kg. GAPDH was served as an internal control. **(I)** Semi-quantitative analysis of the expression of DCX and GFAP in penumbra. **P < 0.01 vs. dMCAO + Vehicle group. Ax, ambroxol; dMCAO, distal middle cerebral artery occlusion; DCX, doublecortin; GFAP, glial fibrillary acidic protein; BrdU, bromodeoxyuridine; ROI, region of interest; DAPI, 4′,6-diamidino-2-phenylindole.

To further understand why administration of 70 mg/kg ambroxol enhances functional recovery and reduces infarct volume, the portion of new born neurons and astrocytes in penumbra, which plays an important role in spontaneous regenerative process to affect infarct volume, was investigated in penumbra following ischemic stroke on day 7. The generation of neuroblasts (DCX^+^) and GFAP^+^ cells were assessed in the SVZ (Figure 2A). The results showed that administration of 70 mg/kg ambroxol increased the number of BrdU^+^&GFAP^+^, while that had no effect on BrdU^+^&DCX^+^ in SVZ (Figures 2B,C). Furthermore, our results demonstrated that portion of GFAP^+^&Nestin^+^ in penumbra was prominently elevated in group of dMCAO + Ax-70 mg/kg, compared to that in group dMCAO + Vehicle (Figures 2D,E). In addition, to evaluate the local neurogenesis, immunostaining in penumbra (Figure 3A) of colocalization of BrdU with DCX or GFAP was performed. The results indicated that the percentage of DCX^+^&BrdU^+^/BrdU^+^ in group dMCAO + Ax-70 mg/kg was significantly increased than that in dMCAO + Vehicle group (Figures 3B,C). And, the portion of GFAP^+^&BrdU^+^/BrdU^+^ in group dMCAO + Ax-70 mg/kg was evidently decreased than that in dMCAO + Vehicle group (Figures 3E,F). While, the percentage of DCX^+^&BrdU^+^/DCX^+^ and GFAP^+^&BrdU^+^/GFAP^+^ showed no obvious difference between group dMCAO + Ax-70 mg/kg, and group dMCAO + Vehicle (Figures 3D,G). In addition, to verify the results collected from immunostaining assays, immunoblotting assays were further performed. The representative bands delineated that the expression of DCX, a marker of immature neuron, was evidently elevated in group dMCAO + Ambroxol-70 mg/kg than that in group dMCAO + Vehicle (Figures 3H,I). Whereas, the expression of GFAP, a marker of astrocyte, was substantially reduced in group dMCAO + Ambroxol-70 mg/kg than that in group dMCAO + Vehicle (Figures 3H,I). Together, these results indicated that administration of 70 mg/kg ambroxol could increase the percentage of DCX^+^ cells, while decrease the portion of GFAP^+^ cells in penumbra, to some degree, induced by ischemic stroke, and finally reduced infarct volume to potentiate functional recovery.

### Administration of 70 mg/kg Ambroxol Up-Regulates β-Glucocerebrosidase (GCase) Expression in Penumbra After dMCAO

**Figure 4:**
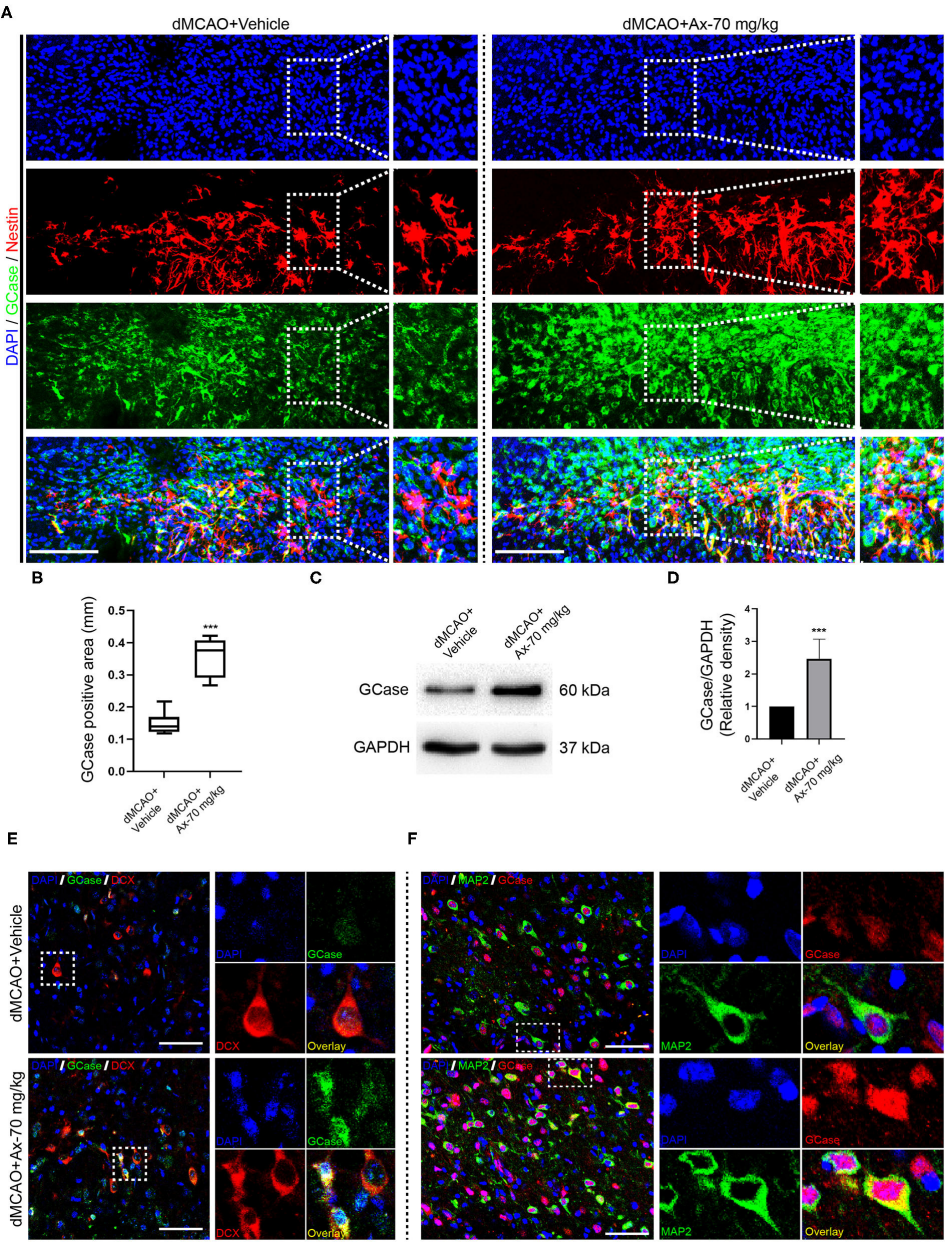
Administration of 70 mg/kg Ax up-regulates GCase expression in penumbra after dMCAO. **(A)** Representative immunostaining images of Nestin and GCase in penumbra post-dMCAO on day 7 in group dMCAO + Vehicle and dMCAO + Ax-70 mg/kg. DAPI was used to counterstain the nuclei. Insets were magnified images from each photograph at high magnification. Scale bar: 100 μm. (**B)** Semi-quantitative analysis of the expression of GCase from **(A)**. ***P < 0.001 vs. dMCAO + Vehicle group. (**C)** Immunoblot bands showing the expression level of GCase in group dMCAO + Vehicle and dMCAO + Ax-70 mg/kg, respectively. GAPDH was served as an internal control. (**D)** Semi-quantitative analysis of GCase expression from **(C)**. ***P < 0.001 vs. dMCAO + Vehicle group. **(E)** Representative immunostaining images of DCX (red) and GCase (green) in penumbra post-dMCAO on day 7 in group dMCAO + Vehicle and dMCAO + Ax-70 mg/kg. DAPI was utilized to counterstain the nuclei. Insets were magnified images from each photograph at high magnification. Scale bar: 20 μm. **(F)** Representative immunostaining images of GCase (red) and MAP2 (green) in penumbra post-dMCAO on day 7 in group dMCAO + Vehicle and dMCAO + Ax-70 mg/kg. DAPI was used to counterstain the nuclei. Insets were magnified images from each photograph at high magnification. Scale bar: 20 μm. Ax, ambroxol; dMCAO, distal middle cerebral artery occlusion; GCase, glucocerebrosidase; DCX, doublecortin.

Previous studies have proven that ambroxol could increase β-glucocerebrosidase (GCase) activity to regulate NSCs differentiation (Migdalska-Richards et al., 2016; Awad et al., 2017; Silveira et al., 2019; Mullin et al., 2020). Hence, we hypothesized that ambroxol could mediate GCase to promote NSCs differentiation into neurons after ischemic stroke, based on above results. Immunostaining was firstly employed to evaluate the expression of GCase in NSCs in penumbra. Our results indicated that administration of 70 mg/kg ambroxol predominantly elevated the optic density not only in NSCs, but also in other cells around penumbra (Figures 4A,B). Next, to assure the results obtained from immunostaining assays, immunoblotting assays were performed. The typical bands represented that the expression of GCase was remarkably increased in group dMCAO + Ambroxol-70 mg/kg than that in group dMCAO + Vehicle (Figures 4C,D). In addition, the expression of GCase in neuronal precursors and mature neurons was also determined using immunostaining. The results showed that administration of 70 mg/kg ambroxol evidently increased the optic density in DCX^+^ neuronal precursors (Figure 4E) and MAP2^+^ mature neurons (Figure 4F). Collectively, these results demonstrated that ambroxol could elevate GCase expression in penumbra, implying that ambroxol and GCapse expression might coordinate together to regulate NSCs differentiation and survival of epibiotic cells in penumbra post-dMCAO.

### Administration of Ambroxol Increases the Expression of GCase and β-Catenin When NSCs Exposed to Oxygen Glucose Deprivation (OGD)

**Figure 5:**
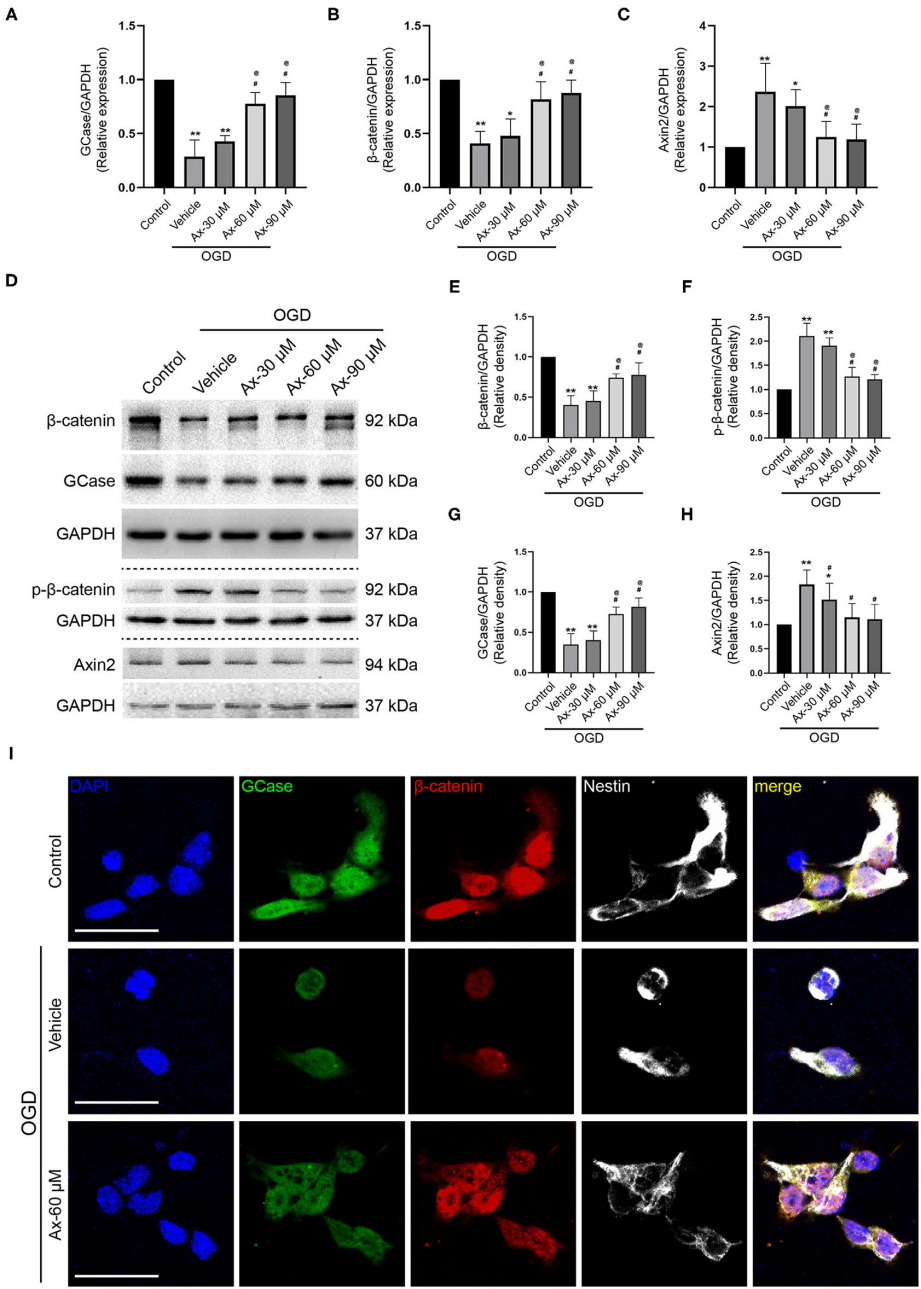
Ax increases the expression of GCase and β-catenin when NSCs exposed to OGD. **(A–C)** Statistical analysis of GCase **(A)**, β-catenin **(B)** and Axin2 **(C)** mRNA expression using RT-qPCR assays in different groups. *P < 0.05, **P < 0.01 vs. control group; ^#^*P* < 0.05 vs. OGD + Vehicle; ^@^*P* < 0.05 vs. OGD + Ax-30 μM. **(D)** Bands depicted the expression of GCase, β-catenin, p-β-catenin and Axin2 in different groups. GAPDH was used as an internal control. **(E–H)** Semi-quantitative analysis of β-catenin **(E)**, p-β-catenin **(F)**, GCase **(G)** and Axin2 **(H)** expression from **(D)**. *P < 0.05, **P < 0.01 vs. control group; ^#^*P* < 0.05 vs. OGD + Vehicle; ^@^*P* < 0.05 vs. OGD + Ax-30 μM. **(I)** Representative immunostaining images of GCase (green), β-catenin (red) and Nestin (white) in various groups. Scale bar: 20 μm. Ax, ambroxol; OGD, oxygen glucose deprivation.

To decipher the potential mechanism of ambroxol promoting NSCs differentiation into neurons, oxygen glucose deprivation (OGD) model was applied to mimic *in vivo* ischemic stroke. Three concentration of ambroxol (30, 60, and 90 μM) was applied to optimize the suitable therapeutic concentration *in vitro* experiments. Firstly, the mRNA expression of GCase, β-catenin and Axin2-a negative mediator of Wnt/β-catenin signaling pathway inducing β-catenin degradation (Jho et al., 2002; Kalani et al., 2008; Fancy et al., 2011), was determined using RT-qPCR assays. The results depicted that OGD could downregulate the expression of GCase and β-catenin, while upregulate the expression of Axin2 (Figures 5A–C). Meanwhile, this effect resulting from OGD could be partially reversed with addition of 60 and 90 μM ambroxol (Figures 5A–C). Moreover, immunoblotting bands represented that OGD could evidently decreased the expression of GCase and β-catenin, compared to Control group (Figures 5D,E,G), and increased expression of p-β-catenin and Axin2 (Figures 5D,F,H). While, administration of ambroxol (60 μM and 90 μM) could partially reverse these protein expressions resulting from OGD (Figures 5D–H). In addition, immunostaining was performed to assess the expression of GCase and β-catenin after NSCs were exposed to OGD for 8 h. The results showed that OGD could markedly decreased the optic density of GCase and β-catenin, compared to Control group (Figure 5I). While, this effect resulting from OGD could be diminished with the application of 60 and 90 μM ambroxol, which was reflected as decreasing the optic density of GCase and β-catenin, to some extent (Figure 5I). Collectively, the results indicated that administration of 60 or 90 μM ambroxol could elevate the expression of GCase and β-catenin and decrease the expression of p-β-catenin and Axin2 after OGD *in vitro*, which was inconsistent with our *in vivo* results. At the same time, the feasible therapeutic dosage of ambroxol used in the present study was 60 μM in our future *in vitro* experiment.

### Wnt/β-Catenin Signaling Pathway Is Involved in Ambroxol Potentiating NSCs Differentiation Into Neurons When NSCs Exposed to OGD

**Figure 6:**
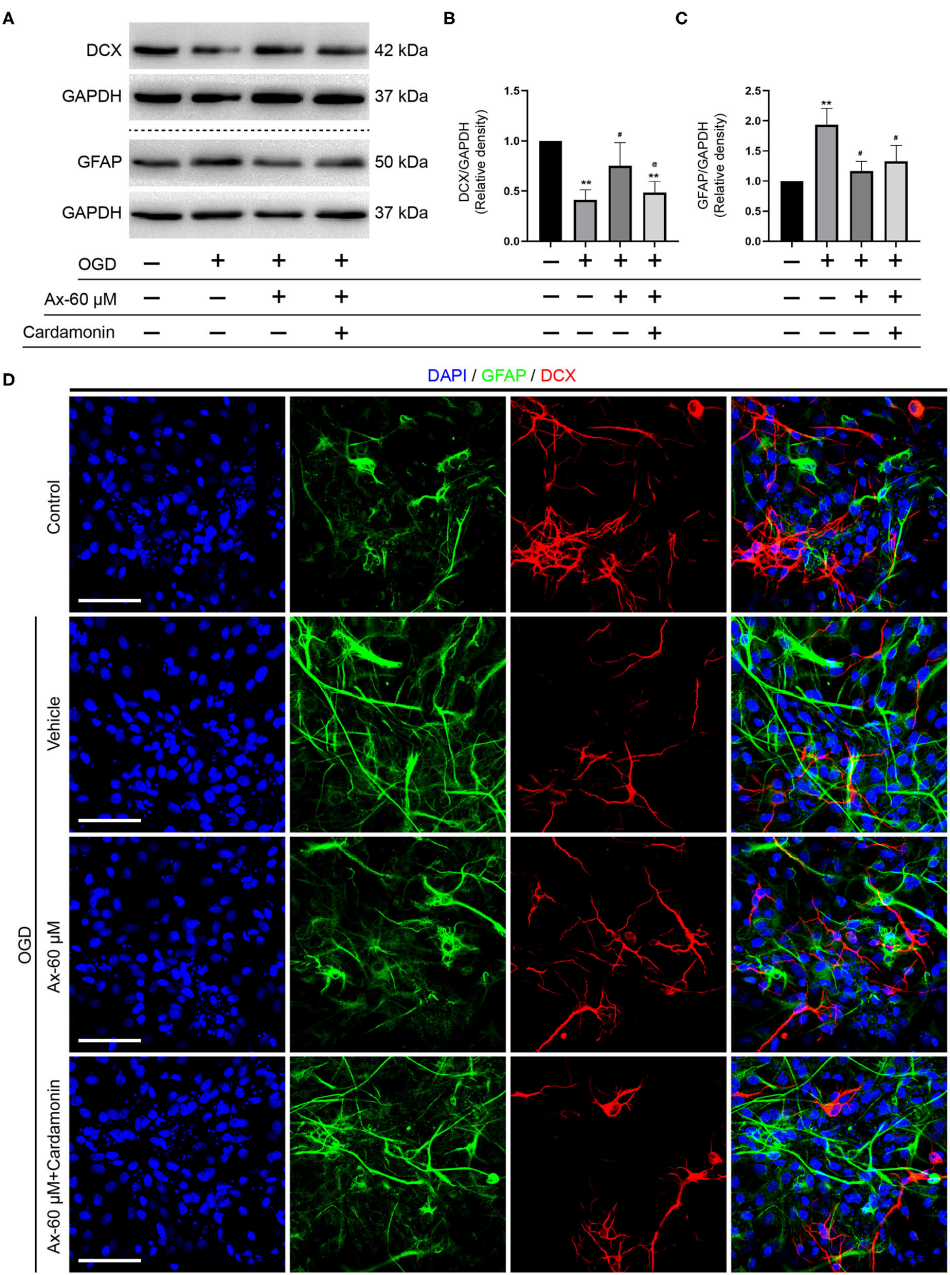
Pharmacological blockage of Wnt/β-catenin signaling pathway partially abrogates the potential of NSCs differentiation into neurons induced by Ax when NSCs exposed to OGD. **(A)** Bands representing the expression of DCX and GFAP in different groups. GAPDH was used as an internal control. **(B,C)** Semi-quantitative analysis of DCX (B) and GFAP **(C)** expression from **(A)**. **P < 0.01 vs. control group; ^#^*P* < 0.05 vs. OGD + Vehicle; ^@^*P* < 0.05 vs. OGD + Ax-60 μM. **(D)** Representative immunostaining images of GFAP (green) and DCX (red) in various groups. Scale bar: 20 μm. Ax, ambroxol; GCase, glucocerebrosidase; OGD, oxygen glucose deprivation; DCX, doublecortin; GFAP, glial fibrillary acidic protein.

**Figure 7:**
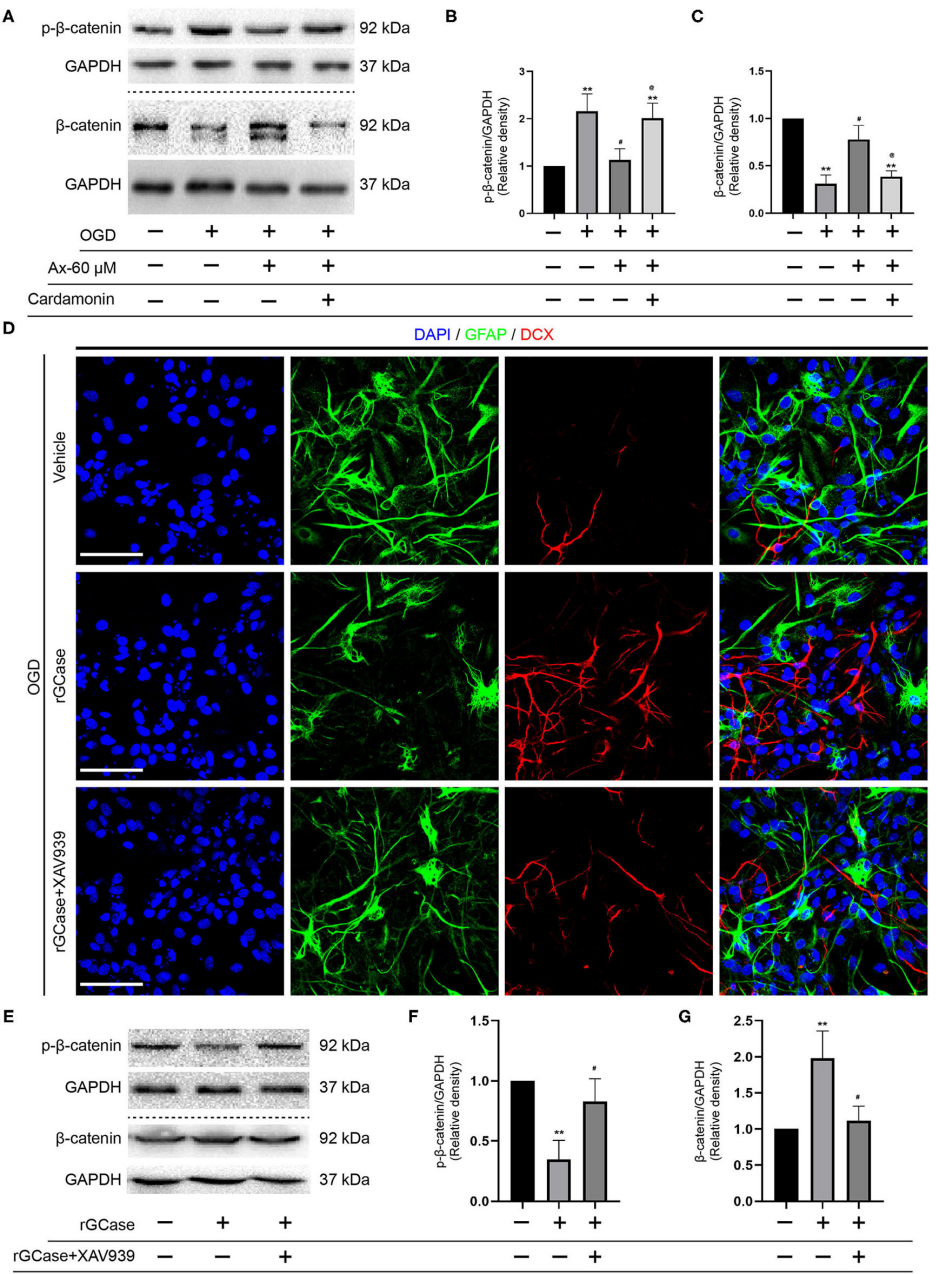
rGCase partially reverses NSCs differentiation into astrocytes induced by OGD through activation of Wnt/β-catenin signaling pathway. **(A)** Bands indicated the expression of p-β-catenin and β-catenin with/without addition of Cardamonin (10 μM). GAPDH was used as an internal control. **(B,C)** Semi-quantitative analysis of p-β-catenin **(B)** and β-catenin **(C)** expression from **(A)**. **P < 0.01 vs. control group; ^#^*P* < 0.05 vs. OGD + Vehicle; ^@^*P* < 0.05 vs. OGD + Ax-60 μM. **(D)** Representative immunostaining images of GFAP (green) and DCX (red) in various groups. Scale bar: 20 μm. **(E)** Bands depicted the expression of p-β-catenin and β-catenin with/without addition of Cardamonin (10 nM). GAPDH was used as an internal control. **(F,G)** Semi-quantitative analysis of p-β-catenin **(F)** and β-catenin **(G)** expression from **(E)**. **P < 0.01 vs. OGD + Vehicle; ^#^*P* < 0.05 vs. OGD + rGCase. Ax, ambroxol; rGCase, recombinant glucocerebrosidase; OGD, oxygen glucose deprivation; DCX, doublecortin; GFAP, glial fibrillary acidic protein.

Above findings suggest that there is a positive correlation between GCase and β-catenin when NSCs were exposed to OGD and with ambroxol administration. Hence, Cardamonin, an inhibitor of Wnt/β-catenin signaling pathway, was applied to investigate the role of Wnt/β-catenin signaling pathway in NSCs differentiation with/without administration of ambroxol. The results revealed that the increased expression of DCX induced by administration of 60 μM ambroxol was obviously decreased with addition of 10 μM Cardamonin (Figures 6A,B). And, the decreased expression of GFAP resulting from administration of 60 μM ambroxol was evidently increased with addition of 10 μM Cardamonin (Figures 6A,C). Next, immunostaining was carried out to attest the results collected from immunoblotting results. The representative images exhibited that 60 μM ambroxol could strengthen NSCs preferred differentiation into neurons, while this enhanced effect was partially abrogated with addition of Cardamonin (Figure 6D). In contrast, the results also revealed that 60 μM ambroxol could attenuated NSCs preferred differentiation into astrocytes, whereas this inhibitory effect was diminished with addition of Cardamonin, to some extent (Figure 6D). Subsequently, the expression of phospho-β-catenin (inactive) and non-phospho-β-catenin (active) was evaluated with/without administration of ambroxol. The bands depicted that Cardamonin effectively abrogated the effect that ambroxol increased the expression of β-catenin and decreased the expression of p-β-catenin, which negatively regulate the accumulation of active β-catenin, and vice versa (Figures 7A–C).

Given that Cardamonin bears the capacity of targeting various signaling molecules, transcriptional factors, cytokines and enzymes, such as mTOR, NF-κB, Akt, STAT3, COX-2, except for Wnt/β-catenin signaling pathway, XAV-93923, a tankyrase inhibitor targeting Wnt/β-catenin signaling, was used to assure the results obtained from Cardamonin. At the same time, the recombinant mouse glucocerebrosidase (10 ng/ml) was used to investigate the correlation between NSCs differentiation and activation of Wnt/β-catenin signaling pathway. The results indicated that rGCase enhanced NSCs differentiation into neurons under OGD condition, which held the similar effect on NSCs differentiation as the ambroxol using immunostaining assays (Figure 7D). Subsequently, the immunoblot bands recapitulated that rGCase effectively increased the accumulation of β-catenin and decreased the expression of p-β-catenin, while XAV939 partially abolished the effect (Figures 7E–G). Together, these data suggest that GCase and β-catenin exhibit a positive correlation, and activating Wnt/β-catenin signaling pathway could direct NSCs differentiation into neurons and inhibit NSCs specification into astrocytes.

## Discussion

Here, our results attested our preconception that administration of ambroxol reduced infarct volume to improve functional recovery in a dose-dependent manner from 35 to 100 mg/kg, and the feasible therapeutic concentration was 70 mg/kg *in vivo*, 60 μM *in vitro*, respectively. Moreover, administration of ambroxol increased GCase expression to activate canonical Wnt/β-catenin signaling pathway resulting in β-catenin accumulation, which promoted NSCs differentiation into neurons, instead of astrocytes, in penumbra after ischemic stroke. Additionally, administration of ambroxol reduced the expression of p-β-catenin and Axin2. The canonical Wnt/β-catenin pathway, one of the highly conserved developmental pathways, plays a pivotal role in various physical and pathological conditions, including regulation of neurogenesis, tissue homeostasis and neural survival in the nervous system (CNS) (Weimar et al., 2002; Marchetti and Pluchino, 2013; Zheng et al., 2017; Marchetti, 2018; Gao et al., 2020). Especially, the homeostasis between unactive state of Wnt/β-catenin pathway and active condition of Wnt/β-catenin pathway dynamically regulates the survival, proliferation, and differentiation of NSCs (Awad et al., 2017; Gao et al., 2020). Inactivation of Wnt/β-catenin pathway induces β-Catenin degradation in the cytoplasm *via* phosphorylation and ubiquitination by glycogen synthase kinase 3 (GSK3) and casein kinase 1 (CK1) (Jho et al., 2002; Gao et al., 2020). While activation of Wnt/β-catenin pathway results in accumulating unphosphorylated β-catenin in the cytoplasm and translocating unphosphorylated β-catenin into the nucleus to exert biological processes (Awad et al., 2017; Gao et al., 2020). Previous studies have revealed that NSCs are partial to differentiation into neurons when unphosphorylated β-catenin cooperates with nuclear transcription factor lymphoid enhancer factor/T cell factor (LEF/TCF) complex (Awad et al., 2017; Marchetti, 2018; Gao et al., 2020), which is in line with our results in the present study. Here, our data indicated that administration of 70 mg/kg ambroxol *in vivo* or 60 μM ambroxol *in vitro* could increase GCase expression to activate Wnt/β-catenin signaling pathway resulting in β-catenin accumulation in cytoplasm, thereafter, potentiating NSCs differentiation into neurons, instead of astrocytes, in penumbra after ischemic stroke. Concurrently, our results also demonstrated that GCase expression was not only upregulated in NSCs, but also increased in other neural cells in penumbra, such as residual neurons, implying that increased β-catenin accumulation in cytoplasm help remanent neural cells survival in penumbra, which is in consistent with previous studies (Shen et al., 2017; Marchetti, 2018). Intriguingly, our results demonstrated that administration of 70 mg/kg ambroxol increased the percentage of DCX^+^&BrdU^+^/BrdU^+^ and decreased the portion of GFAP^+^&BrdU^+^/BrdU^+^, while the percentage of DCX^+^&BrdU^+^/DCX^+^ and GFAP^+^&BrdU^+^/GFAP^+^ had no obvious difference induced by ambroxol, implying that the ratio between neurons and astrocytes remains unchanged, probably owing to the local environmental re-balance post-dMCAO. The neuroinflammation might issue the question that the absolute number of neurons was markedly increased in mice received ambroxol treatment after dMCAO. Previous report has indicated that when the Wnt/β-catenin pathway in the “*Wnt On*” condition, the increased β-catenin in cytoplasm can form a complex with the p50 subunit of nuclear factor kappa-B (NF-κB), which decreases NF-κB transcriptional activity, resulting in microglial polarization from M1 to M2 phenotype and downregulation of inflammation (L'Episcopo et al., 2018). Next, the crosstalk between astrocyte and microglia promotes the release of Wnt1-like proteins in astrocytes, which results in Wnt/β-catenin activation in microglial cells to hamper GSK-3β activation, resulting in a downregulation of proinflammatory mediators (Marchetti et al., 2013; L'Episcopo et al., 2018). Most recently, our research has substantiated that ambroxol enhances neuronal survival and diminishes white matter fiber bundle damage through mitigating M1-like microglial activation and reducing pro-inflammatory cytokines accumulation in mice with ICH (Jiang et al., 2020).

Maintaining dynamic homeostasis of NSCs between proliferation and differentiation is an important mediator to accelerate functional recovery after brain injury. Previous studies have represented that the minority of eNSCs migrated from SVZ differentiate into neurons in penumbra to restore the injured neurovascular network after brain injury (Hermann et al., 2014; Grégoire et al., 2015). Here, mice were injected BrdU to learn the proliferation potential in SVZ and differentiation potential of residuary NSCs in penumbra. Our results showed that mice received 70 mg/kg ambroxol exhibited a larger number of co-labeled BrdU^+^ and GFAP^+^ cells in SVZ, whilst the number of co-labeled BrdU^+^ and DCX^+^ cells showed no visible difference in SVZ. Thereafter, the number of GFAP^+^ and Nestin^+^ cells is evidently elevated in penumbra when mice received ambroxol administration, implying that ambroxol might promote NSCs migration from SVZ to penumbra, that need to be determined in our future research.

Post-stroke pneumonia is a common complication in patients with acute ischemic stroke and always associated with 1-year mortality (de Montmollin et al., 2019). Previous studies have revealed that ambroxol prominimently decreases the bronchopulmonary complications after upper abdominal chest and cardiac surgeries (Yang et al., 2017; Tarrant et al., 2019). Furthermore, study also represents that ambroxol hold the ability of decreasing the rate of lung infection induced by severe cervical spinal cord injury (CSCI) *via* reducing airway secretion and inflammatory factors (Li et al., 2012). Additionally, ambroxol therapy has been assured to be a novel, safe and well-tolerated disease-modifying treatment for Parkinson's disease and Gaucher disease by increasing the GCase activity (Migdalska-Richards et al., 2016; Silveira et al., 2019; Mullin et al., 2020), which is in line with our results in the present study. These evidence offers a clue that ambroxol therapy might be a supplementary treatment for other brain injury. The present study, to our limited knowledge, firstly enlarges the knowledge of ambroxol in regulating NSCs differentiation after ischemic stroke, even in other brain injury.

There are still some limitations need to be sorted out in our next investigation. First, the effect of ambroxol on NSCs migration and the potential mechanism need to be elucidated in our future work. Next, whether other signaling pathway except for canonical Wnt/β-catenin pathway, such as non-canonical Wnt/β-catenin pathway, participates in ambroxol facilitating NSCs differentiation after ischemic stroke needs to be determined. Thereafter, considering that high dosage of ambroxol administration might exert no distinct side effect in a short period in the present study, the long-term side effect needs to be determined in our future research. Meanwhile, the feasible concentration and period of ambroxol administration needs to be determined.

In sum, the present study reveals a novel effect of ambroxol on promoting NSCs differentiation into neurons, while interfering NSCs transformation into astrocytes, through increasing GCase expression to activate Wnt/β-catenin signaling pathway in penumbra after ischemic stroke, which advances basic knowledge of ambroxol in regulating NSCs differentiation and provides a feasible therapy for ischemic stroke treatment, even in other brain disorders in clinic.

## Data Availability Statement

The original contributions presented in the study are included in the article/supplementary materials, further inquiries can be directed to the corresponding authors.

## Ethics Statement

The animal study was reviewed and approved by The Third Military Medical University Ethics Committee (approval no. SYXK 2012-0002).

## Author Contributions

HG performed most of the experiments, with assistance from CZ, YY, WC, JZ, XF, XJ, LT, and YZ. HG, YY, and CZ analyzed the results and edited figures. WC performed dMCAO and statistical analysis. JZ, XF, and LT performed cell culture and treatments. HG, YZ, CZ, and JZ performed immunoblotting and immunostaining. HG wrote preliminary draft of the manuscript. RH and HF edited the manuscript. YC and HF designed experiments and revised the manuscript. All authors approved final version of the manuscript.

## Conflict of Interest

The authors declare that the research was conducted in the absence of any commercial or financial relationships that could be construed as a potential conflict of interest.

## References

[B1] Arredondo S. B. Valenzuela-Bezanilla D. Mardones M. D. Varela-Nallar L. (2020). Role of Wnt signaling in adult hippocampal neurogenesis in health and disease. Front. Cell. Dev. Biol. 8:860. 10.3389/fcell.2020.00860 33042988 PMC7525004

[B2] Awad O. Panicker L. M. Deranieh R. M. Srikanth M. P. Brown R. A. Voit A.. (2017). Altered differentiation potential of gaucher's disease iPSC neuronal progenitors due to Wnt/β-catenin downregulation. Stem Cell Rep. 9, 1853–1867. 10.1016/j.stemcr.2017.10.029 29198828 PMC5785733

[B3] de Montmollin E. Ruckly S. Schwebel C. Philippart F. Adrie C. Mariotte E.. (2019). Pneumonia in acute ischemic stroke patients requiring invasive ventilation: impact on short and long-term outcomes. J. Infect. 79, 220–227. 10.1016/j.jinf.2019.06.012 31238051

[B4] Dillen Y. Kemps H. Gervois P. Wolfs E. Bronckaers A. (2020). Adult Neurogenesis in the subventricular zone and its regulation after ischemic stroke: implications for therapeutic approaches. Transl. Stroke Res. 11, 60–79. 10.1007/s12975-019-00717-8 31309427

[B5] Ding Y. X. Wei L. C. Wang Y. Z. Cao R. Wang X. Chen L. W. (2011). Molecular manipulation targeting regulation of dopaminergic differentiation and proliferation of neural stem cells or pluripotent stem cells. CNS Neurol. Disord. Drug Targets 10, 517–528. 10.2174/187152711795563912 21495963

[B6] Fancy S. P. Harrington E. P. Yuen T. J. Silbereis J. C. Zhao C. Baranzini S. E.. (2011). Axin2 as regulatory and therapeutic target in newborn brain injury and remyelination. Nat. Neurosci. 14, 1009–1016. 10.1038/nn.2855 21706018 PMC3145042

[B7] Gao J. Liao Y. Qiu M. Shen W. (2020). Wnt/β-catenin signaling in neural stem cell homeostasis and neurological diseases. Neuroscientist. 10.1177/1073858420914509. [Epub ahead of print]. 32242761

[B8] Ge H. Tan L. Wu P. Yin Y. Liu X. Meng H.. (2015). Poly-L-ornithine promotes preferred differentiation of neural stem/progenitor cells via ERK signalling pathway. Sci. Rep. 5:15535. 10.1038/srep15535 26503112 PMC4622086

[B9] Ge H. Yu A. Chen J. Yuan J. Yin Y. Duanmu W.. (2016). Poly-L-ornithine enhances migration of neural stem/progenitor cells via promoting alpha-Actinin 4 binding to actin filaments. Sci. Rep. 6:37681. 10.1038/srep37681 27874083 PMC5118728

[B10] George P. M. Steinberg G. K. (2015). Novel stroke therapeutics: unraveling stroke pathophysiology and its impact on clinical treatments. Neuron 87, 297–309. 10.1016/j.neuron.2015.05.041 26182415 PMC4911814

[B11] Grégoire C. A. Goldenstein B. L. Floriddia E. M. Barnabé-Heider F. Fernandes K. J. (2015). Endogenous neural stem cell responses to stroke and spinal cord injury. Glia 63, 1469–1482. 10.1002/glia.22851 25921491

[B12] Hao L. Zou Z. Tian H. Zhang Y. Zhou H. Liu L. (2014). Stem cell-based therapies for ischemic stroke. Biomed Res. Int. 2014:468748. 10.1155/2014/468748 24719869 PMC3955655

[B13] Hermann D. M. Peruzzotti-Jametti L. Schlechter J. Bernstock J. D. Doeppner T. R. Pluchino S. (2014). Neural precursor cells in the ischemic brain - integration, cellular crosstalk, and consequences for stroke recovery. Front. Cell. Neurosci. 8:291. 10.3389/fncel.2014.00291 25278840 PMC4165213

[B14] Huang L. Zhang L. (2019). Neural stem cell therapies and hypoxic-ischemic brain injury. Prog. Neurobiol. 173, 1–17. 10.1016/j.pneurobio.2018.05.004 29758244 PMC6249121

[B15] Jho E. H. Zhang T. Domon C. Joo C. K. Freund J. N. Costantini F. (2002). Wnt/beta-catenin/Tcf signaling induces the transcription of Axin2, a negative regulator of the signaling pathway. Mol. Cell. Biol. 22, 1172–1183. 10.1128/MCB.22.4.1172-1183.2002 11809808 PMC134648

[B16] Jiang X. Zhang J. Kou B. Zhang C. Zhong J. Fang X.. (2020). Ambroxol improves neuronal survival and reduces white matter damage through suppressing endoplasmic reticulum stress in microglia after intracerebral hemorrhage. Biomed Res. Int. 2020:8131286. 10.1155/2020/7941716 32309438 PMC7142346

[B17] Kalani M. Y. Cheshier S. H. Cord B. J. Bababeygy S. R. Vogel H. Weissman I. L.. (2008). Wnt-mediated self-renewal of neural stem/progenitor cells. Proc. Natl. Acad. Sci. U.S.A. 105 16970–16975. 10.1073/pnas.0808616105 18957545 PMC2575225

[B18] Koh S. H. Park H. H. (2017). Neurogenesis in stroke recovery. Transl. Stroke Res. 8, 3–13. 10.1007/s12975-016-0460-z 26987852

[B19] L'Episcopo F. Tirolo C. Serapide M. F. Caniglia S. Testa N. Leggio L.. (2018). Microglia polarization, gene-environment interactions and Wnt/β-catenin signaling: emerging roles of glia-neuron and glia-stem/neuroprogenitor crosstalk for dopaminergic neurorestoration in aged parkinsonian brain. Front. Aging Neurosci. 10:12. 10.3389/fnagi.2018.00012 29483868 PMC5816064

[B20] Li Q. Yao G. Zhu X. (2012). High-dose ambroxol reduces pulmonary complications in patients with acute cervical spinal cord injury after surgery. Neurocrit. Care 16, 267–272. 10.1007/s12028-011-9642-4 22006379

[B21] Marchetti B. (2018). Wnt/β-catenin signaling pathway governs a full program for dopaminergic neuron survival, neurorescue and regeneration in the MPTP mouse model of parkinson's disease. Int. J. Mol. Sci 19:3743. 10.3390/ijms19123743 30477246 PMC6321180

[B22] Marchetti B. L'Episcopo F. Morale M. C. Tirolo C. Testa N. Caniglia S.. (2013). Uncovering novel actors in astrocyte-neuron crosstalk in Parkinson's disease: the Wnt/β-catenin signaling cascade as the common final pathway for neuroprotection and self-repair. Eur. J. Neurosci. 37 1550–1563. 10.1111/ejn.12166 23461676 PMC3660182

[B23] Marchetti B. Pluchino S. (2013). Wnt your brain be inflamed? Yes, it Wnt! Trends Mol. Med. 19, 144–156. 10.1016/j.molmed.2012.12.001 23312954 PMC3595301

[B24] Marchetti B. Tirolo C. L'Episcopo F. Caniglia S. Testa N. Smith J. A.. (2020). Parkinson's disease, aging and adult neurogenesis: Wnt/β-catenin signalling as the key to unlock the mystery of endogenous brain repair. Aging Cell 19:e13101. 10.1111/acel.13101 32050297 PMC7059166

[B25] Migdalska-Richards A. Daly L. Bezard E. Schapira A. H. (2016). Ambroxol effects in glucocerebrosidase and alpha-synuclein transgenic mice. Ann. Neurol. 80, 766–775. 10.1002/ana.24790 27859541 PMC5132106

[B26] Migdalska-Richards A. Ko W. K. D. Li Q. Bezard E. Schapira A. H. V. (2017). Oral ambroxol increases brain glucocerebrosidase activity in a nonhuman primate. Synapse 71:e21967. 10.1002/syn.21967 28295625 PMC5485051

[B27] Mullin S. Smith L. Lee K. D'Souza G. Woodgate P. Elflein J.. (2020). Ambroxol for the treatment of patients with parkinson disease with and without glucocerebrosidase gene mutations: a nonrandomized, noncontrolled trial. JAMA Neurol. 77, 427–434. 10.1001/jamaneurol.2019.4611 31930374 PMC6990847

[B28] Petro M. Jaffer H. Yang J. Kabu S. Morris V. B. Labhasetwar V. (2016). Tissue plasminogen activator followed by antioxidant-loaded nanoparticle delivery promotes activation/mobilization of progenitor cells in infarcted rat brain. Biomaterials 81, 169–180. 10.1016/j.biomaterials.2015.12.009 26735970 PMC4715952

[B29] Poupon-Bejuit L. Rocha-Ferreira E. Thornton C. Hagberg H. Rahim A. A. (2020). Neuroprotective effects of diabetes drugs for the treatment of neonatal hypoxia-ischemia encephalopathy. Front. Cell. Neurosci. 14:112. 10.3389/fncel.2020.00112 32435185 PMC7218053

[B30] Prabhakaran S. Ruff I. Bernstein R. A. (2015). Acute stroke intervention: a systematic review. JAMA 313, 1451–1462. 10.1001/jama.2015.3058 25871671

[B31] Qiu C. W. Liu Z. Y. Zhang F. L. Zhang L. Li F. Liu S. Y.. (2019). Post-stroke gastrodin treatment ameliorates ischemic injury and increases neurogenesis and restores the Wnt/β-catenin signaling in focal cerebral ischemia in mice. Brain Res. 1712, 7–15. 10.1016/j.brainres.2019.01.043 30716287

[B32] Shen Z. Zhou Z. Gao S. Guo Y. Gao K. Wang H.. (2017). Melatonin inhibits neural cell apoptosis and promotes locomotor recovery via activation of the Wnt/β-catenin signaling pathway after spinal cord injury. Neurochem. Res. 42, 2336–2343. 10.1007/s11064-017-2251-7 28417262

[B33] Silveira C. R. A. MacKinley J. Coleman K. Li Z. Finger E. Bartha R.. (2019). Ambroxol as a novel disease-modifying treatment for Parkinson's disease dementia: protocol for a single-centre, randomized, double-blind, placebo-controlled trial. BMC Neurol. 19:20. 10.1186/s12883-019-1252-3 30738426 PMC6368728

[B34] Su X. Li Z. Wang M. Li Z. Wang Q. Lu W.. (2016). The protective effect of different airway humidification liquids to lung after tracheotomy in traumatic brain injury: the role of pulmonary surfactant protein-A (SP-A). Gene 577, 89–95. 10.1016/j.gene.2015.11.024 26611525

[B35] Tarrant B. J. Maitre C. L. Romero L. Steward R. Button B. M. Thompson B. R.. (2019). Mucoactive agents for adults with acute lung conditions: a systematic review. Heart Lung. 48, 141–147. 10.1016/j.hrtlng.2018.09.010 30409442

[B36] Vallée A. Vallée J. N. Guillevin R. Lecarpentier Y. (2018). Interactions between the canonical WNT/beta-catenin pathway and PPAR gamma on neuroinflammation, demyelination, and remyelination in multiple sclerosis. Cell. Mol. Neurobiol. 38, 783–795. 10.1007/s10571-017-0550-9 28905149 PMC11482031

[B37] Van Steenwinckel J. Schang A. L. Krishnan M. L. Degos V. Delahaye-Duriez A. Bokobza C.. (2019). Decreased microglial Wnt/β-catenin signalling drives microglial pro-inflammatory activation in the developing brain. Brain 142, 3806–3833. 10.1093/brain/awz319 31665242 PMC6906599

[B38] Wang Y. Zhao Z. Rege S. V. Wang M. Si G. Zhou Y.. (2016). 3K3A-activated protein C stimulates postischemic neuronal repair by human neural stem cells in mice. Nat. Med. 22, 1050–1055. 10.1038/nm.4154 27548576 PMC5215920

[B39] Weimar C. Roth M. P. Zillessen G. Glahn J. Wimmer M. L. Busse O.. (2002). Complications following acute ischemic stroke. Eur. Neurol. 48, 133–140. 10.1159/000065512 12373029

[B40] Xiong Y. Manwani B. Fisher M. (2019). Management of acute ischemic stroke. Am. J. Med. 132, 286–291. 10.1016/j.amjmed.2018.10.019 30832769

[B41] Xiong Z. G. Zhu X. M. Chu X. P. Minami M. Hey J. Wei W. L.. (2004). Neuroprotection in ischemia: blocking calcium-permeable acid-sensing ion channels. Cell 118, 687–698. 10.1016/j.cell.2004.08.026 15369669

[B42] Yang Y. Zhang K. Chen X. Wang J. Lei X. Zhong J.. (2019). SVCT2 Promotes neural stem/progenitor cells migration through activating CDC42 after ischemic stroke. Front. Cell. Neurosci. 13:429. 10.3389/fncel.2019.00429 31607868 PMC6761321

[B43] Yang Y. Zhang X. Ge H. Liu W. Sun E. Ma Y.. (2018). Epothilone B benefits nigrostriatal pathway recovery by promoting microtubule stabilization after intracerebral hemorrhage. J. Am. Heart Assoc. 7:e007626. 10.1161/JAHA.117.007626 29348323 PMC5850167

[B44] Yang Z. Xiao X. Huang Y. He X. Lu Q. Chen S.. (2017). Effects and mechanisms of ambroxol inhalation (Mucosolvan((R))) in the treatment of neonatal pneumonia. Pharmazie 72, 604–607. 10.1691/ph.2017.7541 29441886

[B45] Zhang D. Lu Z. Man J. Cui K. Fu X. Yu L.. (2019). Wnt-3a alleviates neuroinflammation after ischemic stroke by modulating the responses of microglia/macrophages and astrocytes. Int. Immunopharmacol. 75:105760. 10.1016/j.intimp.2019.105760 31323530

[B46] Zheng H. Jia L. Liu C. C. Rong Z. Zhong L. Yang L.. (2017). TREM2 Promotes microglial survival by activating Wnt/β-catenin pathway. J. Neurosci. 37, 1772–1784. 10.1523/JNEUROSCI.2459-16.2017 28077724 PMC5320608

